# 

**Published:** 1816-04

**Authors:** 


### CORRESPONDENCE.

Dr. Pareira, on certain Endemic Diseases of Rio de Janeiro, will have an early insertion.

Communications have been received from Mr. Gray and Mr. Ellis; which, with a Case of a Wound in the Œsophagus, will appear in our next. The continuation of Dr. Seeds's Experiments is unavoidably delayed, in consequence of the MS. not having come to hand. The last vol. of Med. Chir. Transactions; Hey on Puerperal Fever; and Clarke on Diseases of Females, are under Review.

